# Ectopic Expression of *OsDREB1G*, a Member of the OsDREB1 Subfamily, Confers Cold Stress Tolerance in Rice

**DOI:** 10.3389/fpls.2019.00297

**Published:** 2019-03-28

**Authors:** Seok-Jun Moon, Myung Ki Min, Jin-Ae Kim, Dool Yi Kim, In Sun Yoon, Taek Ryun Kwon, Myung Ok Byun, Beom-Gi Kim

**Affiliations:** ^1^Gene Engineering Division, Department of Agricultural Biotechnology, National Institute of Agricultural Sciences, Rural Development Administration, Jeonju, South Korea; ^2^Metabolic Engineering Division, Department of Agricultural Biotechnology, National Institute of Agricultural Sciences, Rural Development Administration, Jeonju, South Korea; ^3^Crop Foundation Division, National Institute of Crop Science, Rural Development Administration, Wanju-Gun, South Korea; ^4^International Technology Cooperation Division, Technology Cooperation Bureau, Rural Development Administration, Jeonju, South Korea

**Keywords:** rice, OsDREB1s, cold stress tolerance, transgenic plant, transcription factor

## Abstract

Plants adapt to adverse environmental conditions through physiological responses, such as induction of the abscisic acid signaling pathway, stomatal regulation, and root elongation. Altered gene expression is a major molecular response to adverse environmental conditions in plants. Several transcription factors function as master switches to induce the expression of stress-tolerance genes. To find out a master regulator for the cold stress tolerance in rice, we focused on functionally identifying DREB subfamily which plays important roles in cold stress tolerance of plants. Here, we characterized *OsDREB1G* (*LOC_Os02g45450*), a functionally unidentified member of the *DREB1* subgroup. *OsDREB1G* is specifically induced under cold stress conditions among several abiotic stresses examined. This gene is dominantly expressed in leaf sheath, blade, node, and root. Transgenic rice overexpressing this gene exhibited strong cold tolerance and growth retardation, like transgenic rice overexpressing other *OsDREB1* genes. However, unlike these rice lines, transgenic rice overexpressing *OsDREB1G* did not exhibit significant increases in drought or salt tolerance. Cold-responsive genes were highly induced in transgenic rice overexpressing *DREB1G* compared to wild type. In addition, *OsDREB1G* overexpression directly induced the expression of a reporter gene fused to the promoters of cold-induced genes in rice protoplasts. Therefore, OsDREB1G is a typical CBF/DREB1 transcription factor that specifically functions in the cold stress response. Therefore, *OsDREB1G* could be useful for developing transgenic rice with enhanced cold-stress tolerance.

## Introduction

Plant growth and crop productivity are strongly affected by environmental stresses such as drought, salt, and low temperature. To adapt to and survive adverse conditions, a number of genes with diverse functions are induced or repressed in plants under specific stress conditions (Yamaguchi-Shinozaki and Shinozaki, 2005). The expression of stress-responsive genes under abiotic stress is regulated by numerous transcription factors (TF), such as MYB, bZIP, WRKY, AP2/ERF, and bHLH TFs (Shinozaki and Yamaguchi-Shinozaki, 2007; Golldack et al., 2011; Moon et al., 2015). Most stress-related TFs bind to specific *cis-*elements in the promoter regions of numerous stress-inducible downstream genes to activate their transcription, thereby functioning as key regulators of abiotic stress tolerance (Agarwal et al., 2006; Hussain et al., 2011).

One group of transcription factors involved in abiotic stress tolerance is the dehydration responsive element binding (DREB) transcription factor subfamily. DREBs are divided into two subgroups: DREB1 [also referred as C-repeat binding factors (CBFs)] and DREB2. DREB1/CBF and DREB2 transcription factors belong to the ERF subfamily, in the AP2/ERF superfamily with only a single AP2/ERF domain (Nakano et al., 2006; Sharoni et al., 2011; Rashid et al., 2012). DREB transcription factors bind specifically to the DNA sequence motif G/ACCGAC (known as the dehydration responsive element (DRE)/C-repeat; DRE/CRT) found in various cold- and drought-responsive genes (Stockinger et al., 1997; Shinwari et al., 1998). DREB1/CBF TFs primarily function in cold stress, while DREB2 TFs function in drought and salt stress (Liu et al., 1998).

Arabidopsis DREB1/CBF and DREB2 TFs play important roles in abiotic stress responses. *AtDREB1A/CBF3*, *AtDREB1B/CBF1*, and *AtDREB1C/CBF2* are rapidly induced by cold stress treatment, whereas *AtDREB2A* and *AtDREB2B* are induced by drought and high-salinity stress but not by cold stress (Liu et al., 1998). The constitutive expression of *AtDREB1A/CBF1*, *CBF3*, and *CBF4* confers enhanced freezing tolerance in Arabidopsis (Jaglo-Ottosen et al., 1998; Kasuga et al., 1999; Gilmour et al., 2000; Haake et al., 2002). With the completion of the rice (*Oryza sativa* L.) genome project, several studies have described AP2/ERF superfamily and ERF subfamily members in the rice genome. Sharoni et al. (2011) classified the AP2/ERF superfamily into four subfamilies: AP2, RAV, ERF, and DREB. The DREB subfamily including DREB1 and DREB2 consists of 57 genes in rice.

Several *OsDREB1s* have been cloned and phenotypically identified using overexpression transgenic rice among the 11 candidate rice OsDREB1s. The ectopic expression of *OsDREB1A* and *B* enhanced drought, salt, and freezing tolerance in Arabidopsis and rice (Dubouzet et al., 2003; Ito et al., 2006). The overexpression of *OsDREB1D* enhanced cold stress tolerance in Arabidopsis (Zhang et al., 2009). The overexpression of *OsDREB1F*, which is induced by salt, drought, and cold stress, enhanced tolerance to these stresses in both rice and Arabidopsis (Wang et al., 2008). Chen et al. (2008) showed that the constitutive expression of *OsDREB1E* and *OsDREB1G* increased tolerance to water deficit stress in rice. *OsDREB1G* gene (LOC_Os08g43210, GeneBank accession # XM483622) published by Chen et al. (2008) is different from the *OsDREB1G* (*LOC_Os02g45450*) named by several research groups and is equivalent to the OsDREB1I. Mao and Chen (2012) surveyed the gene expression of OsDREB1s and presents the OsDREB1G is induced by cold stress. However, functional analysis of OsDREB1G has not been performed yet. Rice is one of the most important cereal crops worldwide and is used as a model system to study stress-tolerance genes in monocots. Rice is a tropical crop that is sensitive to low temperatures, especially during the reproductive stage (Wang et al., 2003; Imin et al., 2004). Thus, it is important to isolate cold stress tolerance-related genes in rice. In this study, we identified *OsDREB1G*, a novel cold stress-responsive DREB gene in rice, and showed that its overexpression improved cold tolerance in rice. *OsDREB1G* represents a good candidate gene for developing cold stress-tolerant rice.

## Materials and Methods

### Plant Growth Conditions and Stress Treatments

Rice cultivar Dongjin (*Oryza sativa* ssp. Japonica cv. Dongjin) was used in this study. Transgenic rice plants were grown in soil in the greenhouse or on MS [Murashige and Skoog (Duchefa, Netherland)] agar medium [per liter: 4.4 g MS salt, 30 g sucrose, 0.5 g 2-(N-Morpholino) ethanesulfonic acid (MES), 8 g plant agar, pH 5.8] in a growth chamber (EYELA, Japan) maintained at 28°C and 60% relative humidity under long-day conditions (16 h light/8 h dark cycle).

To investigate the expression levels of *OsDREB1G* under various abiotic stress conditions, rice seeds were germinated and grown in soil in a greenhouse. For abiotic stress phenotype analysis, 3-week-old seedlings were treated with osmotic [150 mM mannitol (Sigma-Aldrich, United States)], salt [150 mM NaCl (Sigma-Aldrich, United States)], 5 μM ABA (Sigma-Aldrich, United States) and cold stress (4 C) were treated in MS media. Two-week-old T2 transgenic rice plants were subjected to cold stress (4°C) for 3 days and then recovered for 7 days in a growth chamber maintained at 28°C under long-day conditions (16 h light/8 h dark cycle). The experiments were performed with three biological repetitions. For RNA preparation, 2 week-old seedlings were transferred to a growth chamber at 4°C for cold stress treatment or sprayed with 0.1 mM ABA solution followed by sampling at the designated time points with three biological repetitions.

### RNA Gel Blot Analysis and RT-qPCR

Total RNA was extracted from seedlings and T2 transgenic rice plants with TRIzol Reagent (MRC, United States). Fifteen micrograms of total RNA per lane was electrophoresed on a 1.2% formaldehyde agarose gel and transferred to nylon membranes (Amersham, United Kingdom) by capillary blotting, followed by UV-cross-linking. Pre-hybridization was performed for 30 min at 65°C in Church buffer (1% BAS, 1 mM EDTA, 0.5 M NaHPO4, 7% SDS, pH 7.2), followed by hybridization overnight at 65°C using a *OsDREB1G* probe. The probe was labeled using the random oligonucleotide priming method (Amersham, United Kingdom). The membranes were washed with 2 × SSC with 0.1% SDS for 10 min, followed by 1 × SSC and 0.5 × SSC (with 0.1% SDS) for 10 min each time at 65°C. The washed membrane was exposed to a BAS cassette (Fuji Film, Japan) for 1 day. For quantitative RT-qPCR analysis, first-strand cDNA was synthesized from 4 μg of total RNA using SuperScript III reverse transcriptase (Invitrogen, United States) and then we used the 1/20 volume of total cDNA per RT-qPCR reaction. Amplified signals were detected with a MyiQ real-time PCR system (Bio-Rad, United States) using SYBR Premix Ex Taq^TM^ (Takara, Japan). The amplification parameters were as follows: 5 min of denaturation and enzyme activation at 95°C followed by 40 cycles at 95°C for 5 s, 58°C for 15 s, and 72°C for 20 s. A final step was performed at 65–95°C (1°C/sec) for melting curve analysis. Data were normalized based on the expression of rice *Ubi5*, and relative gene expression was analyzed via the ΔΔCT method. RT-qPCR analysis was performed with at least three biological repetitions. The primer sequences used for RT-qPCR analysis are listed in Supplementary Table S2.

### Subcellular Localization

For subcellular localization, the full-length ORF of *OsDREB1G* without the termination codon was amplified by PCR with specific primers (forward, 5′-TCTAGAATGGACGTTTCTGCTGCGCTC-3′; reverse, 5′-TTGGATCCGTAGCTCCATAGCTGGACCTC-3′). After it was confirmed by sequencing, the amplified fragment was fused to the N-terminus of vector p326GFP under the control of the CaMV35S promoter to create 35S-OsDREB1G:GFP. The fusion construct (35S-*DREB1G:GFP*) was transformed into rice protoplasts, singularly or co-transformed with *NLS-RFP*, the protoplasts were prepared from young etiolated seedlings by PEG-mediated transformation (Kim et al., 2015). To stain the protoplast with DAPI (4′,6′-diamidino-2-phenylindol), after incubating the transformed protoplasts for 24 h at 28°C, protoplasts were fixed by exchanging buffer with W6 solution (154 mM NaCl, 125 mM CaCl_2_, 5 mM KCl, and 10 mM PIPES adjusted to pH 6.8) and 4% paraformaldehyde treatment. After 2 h incubation, the buffer was exchanged by DAPI staining buffer (W6 and 5 ug/ml DAPI). After 10 min incubation and several wash out with W6 solution, DAPI and GFP signals were captured using a Leica TCS SP8 laser scanning confocal microscope. The combinations of excitation wavelength/detection range of emission on the confocal microscopy were 488 nm (solid state laser)/ 493 to 530 bandpass for GFP and 405 nm (solid state laser)/ 410 to 493 bandpass for DAPI. At the co-transformed protoplasts with OsDREB1G-GFP and NLS-RFP, GFP and RFP signals were captured lively by Leica TCS SP8 laser scanning confocal microscope. The excitation wavelength/detection range of emission for RFP was 530 nm (solid state laser)/ 557 to 640 bandpass. The detected images are presented in pseudocolor.

### Transient Transactivation Experiment With Rice Protoplasts

To construct the reporter plasmid vector including the promoter region of *Remorin*, Os03g63870 was fused with the firefly luciferase gene (*fLUC*), and the 1.5 Kb promoter regions of these genes were amplified from Dongjin genomic DNA with specific primers (Supplementary Table S2). The reporter plasmid was constructed using a 135 bp DNA fragment of the Rab16A promoter containing the DRE (GCCGAC) sequence. To construct the effector plasmids, the coding sequences of *OsDREB1A–OsDREB1G* and *OsDREB1G* were cloned into transient expression vector pGEM-3HA containing the maize ubiquitin promoter and a sequence encoding a 3 × HA tag. Freshly isolated rice sheath and leaf protoplasts were co-transfected with the designated reporter (4 μg per transfection) and effector (5 μg per transfection) plasmids. The *UBQ10 promoter::Renilla luciferase* plasmid (0.5 μg per transfection) was added to each sample as an internal control (Kim et al., 2015). Luciferase assays were performed using a dual luciferase assay kit according to the manufacturer’s instructions (Promega, United States). Luciferase assays were performed with at least three biological repetitions.

### Generation of Transgenic Plants and Phenotypic Observation

To generate *OsDREB1G* overexpression rice plants, the coding region of *OsDREB1G* was cloned into the plant expression vector pGA2897 under the control of the maize *UBIQUITIN* promoter (Kim et al., 2012). Recombinant DNA containing *OsDREB1G* was introduced into *Agrobacterium tumefaciens* LBA4404 by electroporation using a MicroPulser Electroporator (BioRad, United States). Transgenic rice plants were obtained by Agrobacterium-mediated transformation as previously reported (Han et al., 2012). Transgenic T0 plants were selected on medium containing hygromycin, transferred to soil in pots, and grown in a greenhouse. Transgenic plants overexpressing *OsDREB1G* were chosen for further study via RNA gel blot and RT-qPCR analysis. To investigate the effect of *OsDREB1G* on cold stress tolerance, 7 *OsDREB1G*-overexpressing plants (including independent lines #7, #8, #12, #42) and wild-type plants were grown together in the same pots filled with soil for 2 weeks in the greenhouse. The plants were transferred to a cold chamber (EYELA, Japan) at 4°C for 3 days, after which they were grown for 7 days under normal conditions (28°C, 16 h light/8 h dark cycle). Cold stress tolerance was assessed by determining the survival rate during the recovery period. And then four independent lines (including independent lines #7, #8, #12, #42) were selected to cold stress analysis in T2 generation using same method.

### Post-germination Assay

For the post-germination assay, sterilized non-transgenic and transgenic rice seeds were planted on 1/2MS medium with or without 40 mg/L hygromycin. Three days after planting, the plants were transferred to 1/2MS medium with or without 5 μM ABA (2-*cis*-4-*trans*-abscisic acid, 98% synthetic). Seedling growth was measured at 7 days after transfer.

## Results

### *OsDREB1G* Is Specifically Expressed During Cold Stress

**Figure 1:**
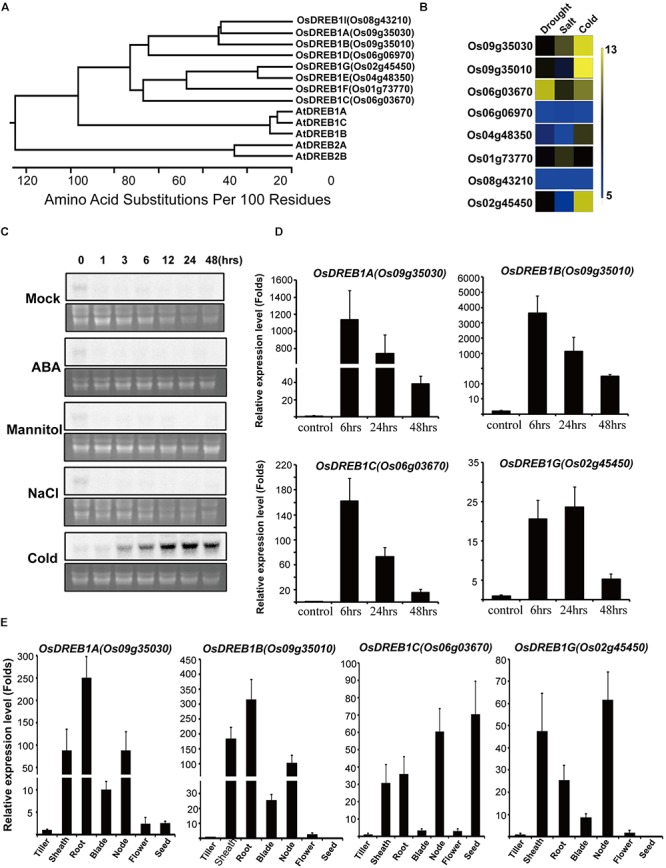
*OsDREB1G* is specifically expressed in response to cold treatment. **(A)** Phylogenetic analysis of *OsDREB1s*. The phylogenetic tree was constructed based on the deduced amino acid sequence of *OsDREB1* and other AP2 domain protein sequences using the DNASTAR program (DNASTAR, Inc., Madison, WI, United States). **(B)** Gene expression analysis of several OsDREB1s under different abiotic stress treatments using genevestigator microarray database (GSE6901). **(C)** *OsDREB1* is specifically upregulated in response to cold stress treatment. Total RNA was isolated from 2-week-old rice plants treated with ABA (100 μM), mannitol (150 mM), NaCl (150 mM), or cold (4°C). A gel pre-stained with ethidium bromide (lower panels) was used to confirm equal loading in all wells. **(D)** Gene expression analysis of *OsDREB1s* in several time intervals under cold treatment. RNA was isolated from 2-week-old plants grown in 1/2MS media. **(E)** Gene expression analysis of *OsDREB1Gs* in different tissues. RNA was isolated from the tissues of 1 or 2-month-old plants. Error bars indicate standard errors. The experiments were performed with at least three biological repetitions.

Among *OsDREB1s*, only six members have been characterized functionally in terms of their effects on phenotype and genetics. Thus, to functionally characterize the other members of the *OsDREB1* gene family, we searched the microarray database^1^ to identify *OsDREBs* that are responsive to cold stress. We found that Os02g45450 was specifically expressed in response to cold stress, with an expression profile similar to those of *OsDREB1A* and *OsDREB1B* (Figure 1B). Os02g45450 was closest *to OsDREB1E* and *OsDREB1F* in a phylogenic tree constructed based on amino acid sequences (Figure 1A). This gene was identified as *OsDREB1G* and studied only about gene expression profiles under abiotic stress conditions. We summarized OsDREB1 subgroup genes published (Supplementary Table S1) We confirmed its expression pattern by RNA gel blot analysis in plants under several stress conditions (Figure 1C). Cold treatment increased *OsDREB1G* expression at 3 h, with expression levels reaching a maximum at 24 h and remaining quite high until 48 h after treatment. However, the expression of *OsDREB1G* did not change in response to other stress treatments including NaCl, mannitol, and abscisic acid (ABA) treatment (Figure 1C). We also compared the *OsDREB1G* expression pattern with those of other *OsDREB1* genes using reverse transcription quantitative polymerase chain reaction (RT-qPCR) (Bustin et al., 2009). Cold stress induction of *OsDREB1G* was weaker than that of other OsDREB1 genes, although *OsDREB1G* had a longer induction period. *OsDREB1G* maintained high expression until 24 h after cold treatment (Figure 1D).

We compared the expression of this gene in several tissues to that of other *OsDREB1* genes using RT-qPCR. *OsDREB1G* was strongly expressed in root, sheath, node, and leaf blade tissue and expressed in tiller and flower at low level. However, *OsDREB1G* was almost never expressed in seeds. This expression pattern was quite similar to those of *OsDREB1A* and *OsDREB1B* (Figure 1E).

### *OsDREB1G* Is a Typical Transcription Factor That Binds to DRE Elements

**Figure 2:**
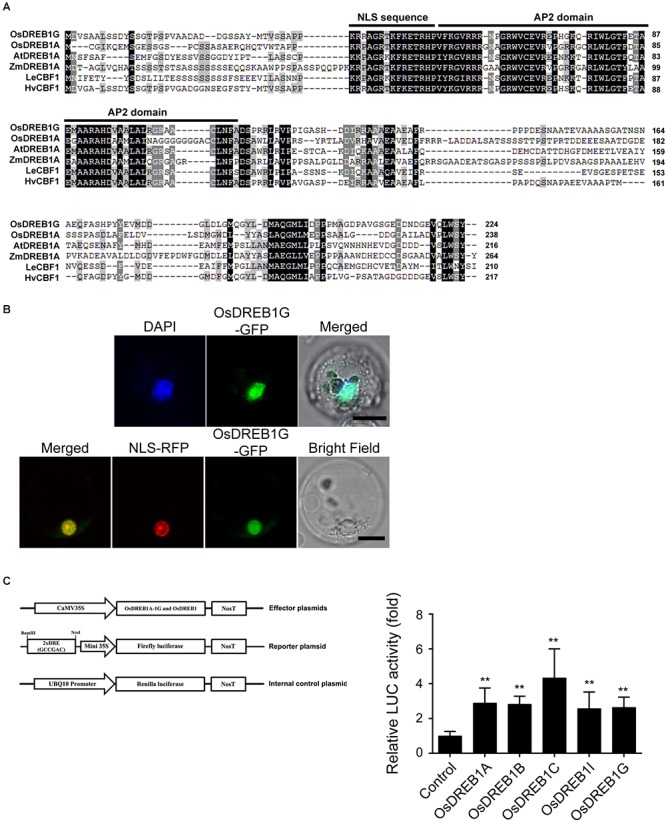
OsDREB1G has typical characteristics of OsDREB1 proteins. **(A)** Multiple sequence alignment of OsDREB1G and homologous proteins **(B)** Subcellular localization of OsDREB1G-GFP fusion protein in rice protoplasts. The protoplasts were transformed with only *OsDREB1G* or *NLS-RFP* (nucleus marker) together and then for 24 h incubation at 28°C, the protoplasts were observed (lower panel) and after DAPI staining by laser scanning confocal microscope (upper panel). Scale bars are 10 mm. **(C)** Transactivation analyses of several OsDREB1s for the DRE element. Luciferase activity was measured using rice protoplasts transfected by each effectors and reporter. Error bars indicate standard errors. The experiments were performed with at least three biological repetitions. ^∗∗^means *P* < 0.01.

The coding region of *OsDREB1G* is 675 bp and encodes a putative 224 amino-acid protein with a predicted molecular mass of 23.9 kDa (GenBank Accession No. XP_015624757, TIGR ID LOC_Os02g45450, RAP ID Os02g0677300). OsDREB1G contains a putative nuclear localization signal (NLS), a conserved AP2/ERF DNA binding domain, and an LWSY motif (Figure 2A). To investigate whether OsDREB1G is a functional transcription factor, we confirmed the subcellular localization of OsDREB1G protein. A fusion construct containing *OsDREB1-GFP* driven by the CaMV35S promoter was introduced into rice protoplast cells. The OsDREB1-GFP fusion protein was co-localized to the nucleus with NLS-RFP and DAPI stain, indicating that OsDREB1G is a nuclear protein (Figure 2B). DREB1s are well-known transcription factors that regulate gene expression by binding to the CRT/DRE sequences of stress-responsive downstream genes (Sakuma et al., 2002). We performed a transactivation assay of OsDREB1G compared to other OsDREB1s in rice protoplasts. The luciferase gene (*LUC*) fused to 2 × DRE (GCCGAC) and the 35S minimal promoter was used as a reporter plasmid. Relative LUC activity increased in the presence of all OsDREB1s in rice protoplasts. OsDREB1G, OsDREB1A, and OsDREB1B showed similar transactivation activities, while OsDREB1C had the highest transactivation activity (Figure 2C). These results indicate that OsDREB1G is a typical member of the OsDREB1 subgroup.

### *OsDREB1G*-Overexpressing Transgenic Rice Shows Enhanced Cold-Stress Tolerance

**Figure 3:**
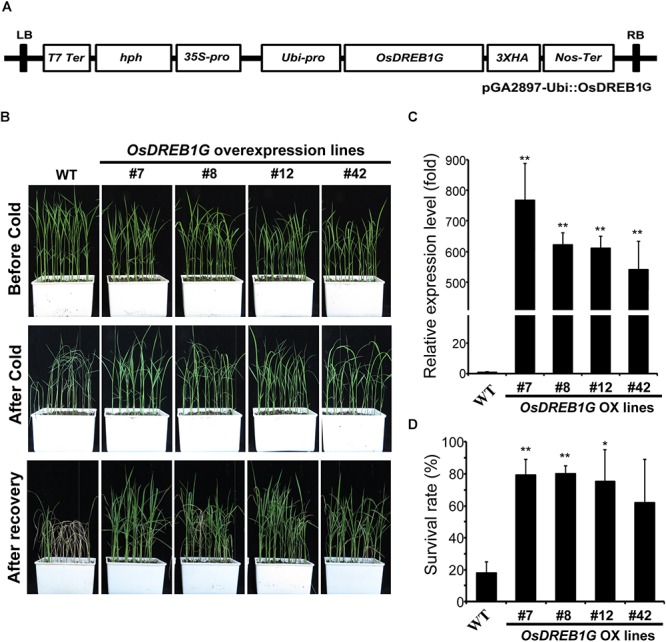
*OsDREB1G*-overexpressing transgenic rice shows enhanced cold stress tolerance. **(A)** Vector map for overexpress *OsDREB1G* and transform into rice. **(B)** Two-week-old T2 transgenic rice plants were subjected to cold stress (4°C) for 3 days and then recovered for 7 days in a growth chamber maintained at 28°C and 60% relative humidity under long-day conditions (16 h light/8 h dark cycle). **(C)** Gene expression analysis of *OsDREB1G* in four independent T2 *OsDREB1G* overexpression transgenic rice lines by RT-qPCR. RNA was isolated from two-week-old seedlings grown in 1/2 MS media. **(D)** Survival rates of transgenic rice plants overexpressing *OsDREB1G* under cold-stress conditions. Error bars indicate standard errors. The experiments were repeated three times. ^∗^means *P* < 0.05, ^∗∗^means *P* < 0.01.

To determine whether the overexpression of *OsDREB1G*, like other *OsDREB1*s, would improve abiotic stress tolerance, we constructed and transformed the vector for overexpression into rice (Figure 3A) and we examined cold, drought, salt, and ABA tolerance in *OsDREB1G* overexpression (OsDREB1G-Ox) plants. We generated 42 transgenic rice plants, investigated the expression of *OsDREB1G* in these plants, and selected four *OsDREB1G*-Ox lines (#8, #12, #42) for further studies based on the gene expression level and confirmed the over-expression of selected lines using RT-qPCR (Figure 3C). Among the various stress treatments, *OsDREB1G*-OX plants showed clearly enhanced cold-stress tolerance compared to the control in the T2 generation (Figure 3B). Under cold-stress treatment, the control plants (Dongjin) showed a survival rate of 20%, whereas all four *OsDREB1G*-OX lines showed survival rates >60% (Figure 3D). However, *OsDREB1G*-OX plants were not sensitive to ABA in terms of growth (Supplementary Figure 1), and they did not exhibit clear and reproducible changes in drought or salt tolerance (Supplementary Figure 2). Finally, under normal growth conditions, the *OsDREB1G*-OX plants had shorter stems and lower seed productivity compared to the control (data not shown).

### The Overexpression of OsDREB1G Induces Cold-Specific Marker Gene Expression and Transactivates the Promoters of These Genes in Rice

**Figure 4:**
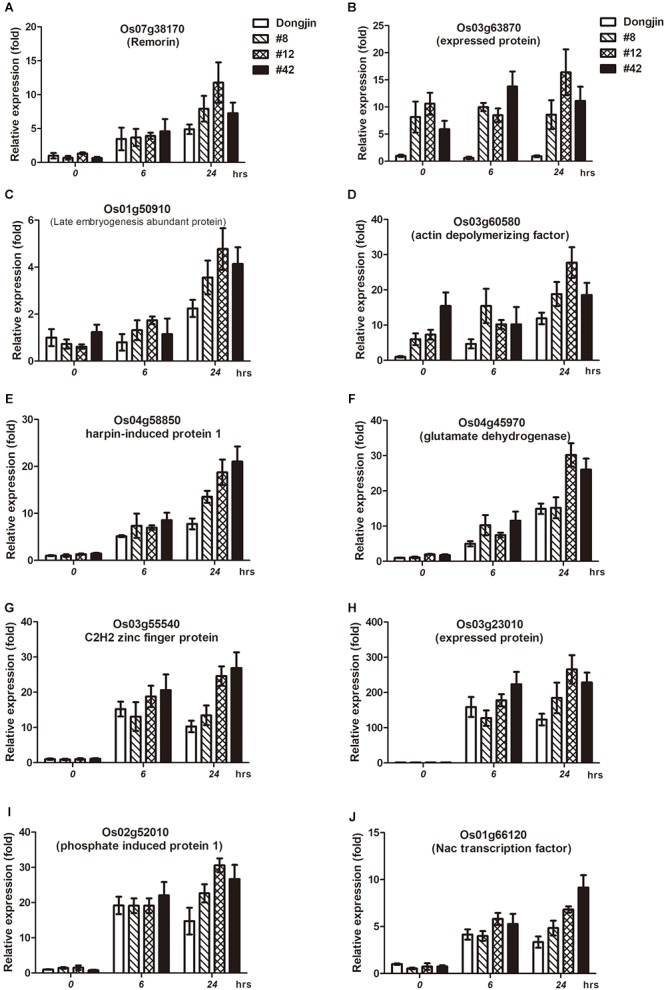
Transgenic rice overexpressing *OsDREB1G* shows enhanced expression of cold-stress marker genes. (**A**–**J)**, Gene expression analysis of putative cold stress-responsive genes in T2 transgenic rice plants overexpressing *OsDREB1G* under cold stress condition. RNA was isolated from 2-week-old plants treated with cold (4°C) in different time intervals. All RT-qPCR were performed using same conditions. Error bars indicate standard deviations. The experiments were performed with at least three biological repetitions.

We performed expression analysis of cold stress-responsive genes in *OsDREB1G*-OX plants. Firstly we selected 16 cold responsive genes published by two research groups and unpublished 3 genes we have information in my laboratory (Rabbani et al., 2003; Byun et al., 2015). And then we examined the expression levels of them by RT-qPCR in *OsDREB1G*-Ox plants and we found out that 10 genes of them were upregulated in three different OsDREB1G overexpression lines and different time points of cold stress treatment compared to control. These genes were expressed at levels up to ∼3-times higher in *OsDREB1G*-Ox plants compared to the control under cold treatment, and Os03g60580 was expressed at higher levels in *OsDREB1G*-Ox plants, even at normal temperatures (Figure 4A–J). Thus, cold stress-tolerance genes were more highly expressed in *OsDREB1G*-OX plants than the control, suggesting that these plants have enhanced cold tolerance.

**Figure 5:**
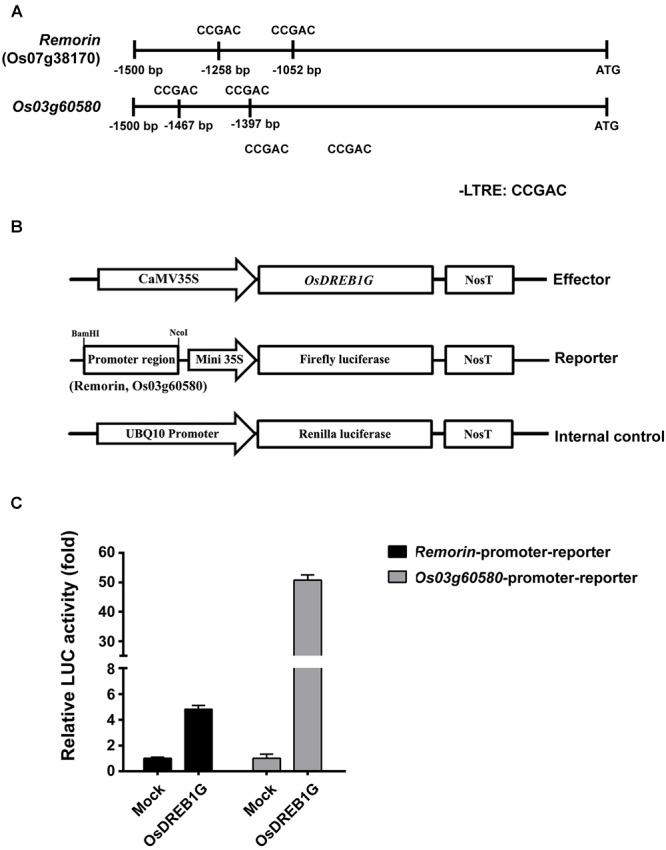
Transactivation of cold-inducible promoter-luciferase fusion genes by OsDREB1G. **(A)** Promoter analysis of the target genes *Remorin* and *Os03g60580*. ATG indicates transcription start site. Putative CRT/DRE motifs are indicated in each promoter region. **(B)** Schematic diagram of the effector and reporter constructs used for co-transfection. **(C)** *OsDREB1G* was transfected with each *promoter-fLUC* reporter plasmid and *UBQ10-rLUC* plasmid as an internal control into rice protoplasts by PEG transfection. Error bars indicate standard errors. The experiments were performed with at least three biological repetitions.

To investigate whether OsDREB1G directly induces the expression of these marker genes, we produced reporter constructs in which the promoters of the cold-stress marker gene *Remorin* and of *Os03g60580* were individually fused with the luciferase reporter gene. We co-transformed rice protoplasts with an *OsDREB1G* effector construct and the reporter construct, finding that the *Remorin* promoter was induced at a level approximately 5-fold higher than the control, whereas the *Os03g60580* promoter was induced at a level >50-fold higher than the control. Indeed, gene expression analysis showed that *Os03g60580* was more highly induced in *OsDREB1G*-OX plants in response to cold treatment than *Remorin* (Figure 5). These results indicate that *OsDREB1G* directly induces cold-responsive gene expression in rice.

## Discussion

DREB1/CBFs are transcription factors that confer cold, drought, and salt tolerance to plants by regulating the expression of genes containing DRE/CRT elements, as revealed in Arabidopsis using genetic approaches (Stockinger et al., 1997; Shinwari et al., 1998). After the completion of genome sequencing of Arabidopsis, rice, and other plant species, the DREB genes were systematically identified as a subfamily of the AP2/ERF superfamily in plants (Skinner et al., 2005; Wu et al., 2015; Zandkarimi et al., 2015; Chen et al., 2016; Dossa et al., 2016; Li et al., 2017). More than 50 genes have been classified as DREB subfamily genes in rice by various research groups (Nakano et al., 2006; Sharoni et al., 2011; Rashid et al., 2012). Even though several *OsDREB1s* genes have been functionally identified, the functions of many *OsDREBs* have not been determined (Dubouzet et al., 2003; Ito et al., 2006; Wang et al., 2008; Zhang et al., 2009; Mao and Chen, 2012). Thus, it remains unclear why *OsDREBs* have so many functional homologs and how the functions of many OsDREBs are regulated via a complex network. In this study we found that the transactivity and *cis-*element binding ability of OsDREB1G is similar to other OsDREB1s such as OsDREB1A, B even though OsDREB1C has much higher activity. OsDREB1G shares the highest amino acid sequence similarity with OsDREB1E and OsDREB1F. Thus we expected that the overexpression of the gene improved the drought, salt and cold stress tolerance like other OsDREB1s. However, *OsDREB1*G-OX rice exhibited only cold stress tolerance rather than drought or salt tolerance. OsDREB1G is specifically expressed under cold stress condition and showed no response to ABA, osmotic stress and salt stress. And it is dominantly expressed in leaf like OsDREB1A and OsDREB1B. Also, the gene expression of *OsDREB1A*, *B,* and *C* were induced at maximum level in 6 h after cold treatment but the gene expression of *OsDREB1G* started to increase from 6 h and reached at maximum in 24 h. Under cold stress, the expression level of cold stress marker genes showed clear differences after 24 h between control and transgenic rice overexpressing OsDREB1G except for two genes (Os03g63870, Os03g60580). The induction level of OsDREB1G gene expression was also quite lower than other OsDREB1s in cold treatment condition. Taken together, OsDREB1G might be not initially and rapidly responsive transcription factor for cold stress and might evoke secondary and prolonged responses in cold stress tolerance.

Arabidopsis *CBF1*, *CBF2*, and *CBF3* are tandemly located on chromosome 4. In rice, *OsDREB1A (Os09g35030)*, *OsDREB1B (Os09g35010),* and *OsDREB1H (Os09g35020)* are tandemly located on chromosome 9. Thus, these genes might have duplicated before the divergence of monocots and dicots. Two other *OsDREB1* genes [*OsDREB1I* (*Os08g43200*) and *OsDREB1J* (*Os08g43210*)] are present in tandem on chromosome 8 (Mao and Chen, 2012). The tandem repeat on chromosome 8 might have arisen after divergence of monocot and dicot. As mentioned it, the members of rice OsDREB1 subgroup have high redundancy and amino acid homology among them. Thus, knock-out or knock-down rice of single gene might present hardly abiotic stress related phenotypes because of the complementation of other homologous OsDREB1 genes. So far, knockout or knockdown rice of OsDREB1s has not been reported yet. Thus we did not try to make knock-out or knock-down rice of OsDREB1G and made only the transgenic rice to overexpress OsDREB1G to dissect the functions of the gene.

Dehydration responsive element TACCGACAT, C-repeated (CRT) GGCCGACAT, and low temperature-responsive element (LTRE) GGCCGACGT are the binding sites for *OsDREB1s*. These elements share the consensus sequence CCGAC. DREB1s have different binding affinities for DRE elements due to differences in two bases (G/A and NA/G/C) located near both ends of the consensus sequence. The OsDREB1s also showed different transactivation activities for DREs. The distance of these *cis-*elements from the transcription start site also affects their transcriptional activity (Sakuma et al., 2002; Yamaguchi-Shinozaki and Shinozaki, 2005; Yang et al., 2009). Thus, these variations of *cis-*acting element binding activity of DREBs and several kinds of DREBs can make many combinations to elaborately regulate gene expression in response to the different strengths and kinds of environmental stresses. Functional studies for each OsDREB1s will shed light on the complicated transcriptional regulatory networks underlying abiotic stress responses.

In Arabidopsis, several upstream genes regulate the expression of *DREB1s*. Among these, ICE1 is a master regulator of *CBF* gene expression. ICE1 protein stability is negatively and positively regulated by phosphorylation via MAPK3/6 and OST1, respectively (Ding et al., 2015; Zhao et al., 2017). These signaling components are conserved in rice, and similar molecular mechanisms might also have been conserved during evolution, like other molecular mechanisms involving OsDREB1s (Kim et al., 2012; Deng et al., 2017). Investigating how OsDREB1s are regulated in response to environmental stress by upstream signaling components and how the OsDREB1s regulate target gene expression and form complex gene expression networks through several different DREB1s will facilitate the development of cold stress-tolerant crops.

## Author Contributions

S-JM cloned the all genes, gene expression analysis, and performed the cold stress tolerance. MM carried out the luciferase assay and organized the figures. J-AK managed all plant materials and did RT-qPCR analysis. DK designed this project with MB and performed the rice transformation. IY revised the manuscript and designed the project. TK and MB designed this research project as previous team leader and supervised the project and managed the transgenic rice. B-GK and S-JM wrote the manuscript, analyzed the data, and designed the experiments. All authors read and approved the manuscript.

## Conflict of Interest Statement

The authors declare that the research was conducted in the absence of any commercial or financial relationships that could be construed as a potential conflict of interest.

## Footnotes

1 https://genevestigator.com
