# Supplementary material for: Ectopic Expression of OsDREB1G, a Member of the OsDREB1 Subfamily, Confers Cold Stress Tolerance in Rice
Source: Front Plant Sci. 2019 Mar 28;10:297. doi: 10.3389/fpls.2019.00297 (PMC6447655; doi:10.3389/fpls.2019.00297)
Supplement: Supplementary file 1 [file Table_1.DOCX]

Supplementary Table 1.List of OsDREB1 subfamily genes published

| MSU ID | RAP-ID | GeneBank ID | Gene Name | Characteristics |
| --- | --- | --- | --- | --- |
| Os09g35030 | Os09g0522200 | AF300970^1,2^ | OsDREB1A^1,2^ | Cold, drought and salt tolerance |
| Os09g35010 | Os09g0522000 | AF300972^1^,AY785894^2^ | OsDREB1B^1,2^ | Cold, drought, and salt tolerance |
| Os06g03670 | Os06g0127100 | AP001168 (nucleotide 142337–142981)^1,2^ | OsDREB1C^1,2^ | Cold, drought and salt tolerance |
| Os06g06970 | Os06g0165600 | AB023482 (nucleotide 1489–2250)^1^,AY785895^2^ | OsDREB1D^1,2^ | Cold and salt tolerance |
| Os04g48350 | Os04g0572400 | AY785896^2,3,5^ | OsDREB1E^,2,5^ | Mild drought tolerance |
| Os01g73770 | Os01g0968800 | AY785897^2,5^,AY345234^4^ | OsDREB1F^2,4,5^ | Salt, drought and low temperature tolerance |
| Os02g45450 | Os02g0677300 | AP005775 (nucleotide 57339-58013) ^2,5^ | OsDREB1G^2,5^ | cold responsive gene expression |
| Os09g35020 | Os09g0522100 | AAAA01001957^2,5^ | OsDREB1H^2,5^ |  |
| Os08g43210 | Os08g0545500 | AP004632 (136639-137349)^2,5^, XM483622^3^ | OsDREB1I^2,5^ OsDREB1G^3^ | Drought tolerance |
| Os08g43200 | Os08g0545500 | AP004632(140485-141973)^2,5^ | OsDREB1J^2,5^ |  |

1. Dubouzet, J. G.; Sakuma, Y.; Ito, Y.; Kasuga, M.; Dubouzet, E. G.; Miura, S.; Seki, M.; Shinozaki,

K.; Yamaguchi-Shinozaki, K., OsDREB genes in rice, Oryza sativa L., encode transcription activators

that function in drought-, high-salt- and cold-responsive gene expression. *Plant J* **2003,** *33* (4), 751-63.

2. Jeffrey S. Skinner, Jarislav von Zitzewitz, Pe´ter Sz}ucs, Luis Marquez-Cedillo,Tanya Filichkin, Keenan Amundsen, Eric J. Stockinger, Michael F. Thomashow,Tony H.H. Chen1, Patrick M. Hayes Structural , functional and phylogenetic characterization of a large CBF gene family in barley. Plant molecular bilogy 2005,59, 533-51

3.Jian-Qiang Chen, Xiu-Ping Meng, Yun Zhang, Mian Xia, Xi-Ping Wang, Over-expression of OsDREB genes lead to enhanced drought tolerance in rice. Biotechnology Lett 2008, 30,2191-2198

4 Qiuyun Wang, Yhcheong Guan, Yaorong Wu, Honglin Chen, Fan Chen, Chengcai Chu, Overexpression of a rice OsDREB1F gene increases salt, drought, and low temperature tolerance in both Arabidopsis and rice. 2008 Plant Mol. Biol 67,589-602

5. Mao, D., and Chen, C. (2012). Colinearity and Similar Expression Pattern of Rice DREB1s Reveal Their Functional Conservation in the Cold-Responsive Pathway. PLoS One 7.
