# Supplementary material for: Ectopic Expression of OsDREB1G, a Member of the OsDREB1 Subfamily, Confers Cold Stress Tolerance in Rice
Source: Front Plant Sci. 2019 Mar 28;10:297. doi: 10.3389/fpls.2019.00297 (PMC6447655; doi:10.3389/fpls.2019.00297)
Supplement: Supplementary file 2 [file Table_2.DOCX]

Supplementary Table 2 . Primer sequences used for quantitative real-time PCR

|  | Forward(5’-3’) | Reverse(5’-3’) |
| --- | --- | --- |
| Remorin  (LOC_Os07g38170) | AACGCCTGGCTCAAGAAGTA | ACGGCCCTCATGAAGTTGG |
| Os03g63870 | TAACCCACAGGTGGTCAGGA | GCAGGAACCAAGTCCCTGAT |
| Os10g22630 | CCCATCGTCGGTAGTCACTG | AGGAGCCACAGGAGATGCTA |
| Os11g34790 | TGGTTCAGAGCTCTTGCTGG | AACAAGGCGTGGTCTCCAAT |
| OsDREB1G  (Os02g45450) | CCACTAATTCGAACGCCGAAC | GCTACCTACGCAGGATCAC |
| OsDREB1A | GACGTCCTGAGTGACATGGG | AGTAGCTCCAGAGTGGGACG |
| OsDREB1B | CTCGCACTGAAAAGTGTGGAC | GGAGGGAGAAATCTGGCACA |
| OsDREB1C | TCTGGAGCTACTGATGATCGC | AGCTGTATAGGAGGAGCAAAGC |
| Os01g50910 | AGGGGAGCAGGTGAAGAG | TGTAGGTGCTGGTGTCCTT |
| Os01g66120 | CGGGTCGGGGTACTGGAA | AGTTGGTCTTCTCGCCCTTG |
| Os02g52010 | ATCGTAGGGTTTGCACCGTC | TGTGGTACGTGAGTTGCTCG |
| Os03g55540 | GTTCGACCTCAACCTCCCG | TTACGCGGTGAGAAGCCG |
| Os04g58850 | CGACATCGAGGTCAAGCTCA | CAGACTTTGCAGTCGGTGC |
| Os04g45970 | GTGGAGTGCTCAACAGGGAA | ACCGGAGTTGGCGTAAATGT |
| Os03g23010 | TCTCCCATGTGTGTGTGCAG | TACTTGGTGGGCTTCTCGGA |
| Os03g60580 | GTGAGGAGCAAGATGGTGT | GAGGTGTGGTCCTTGAGC |
| Ubi5 | CGCCGTGCTCCAGTTCTACAAGG | TCCTTCCTTACTTCCGCCCCCA |
