# Supplementary figures and images for: Ectopic Expression of OsDREB1G, a Member of the OsDREB1 Subfamily, Confers Cold Stress Tolerance in Rice
Source: Front Plant Sci. 2019 Mar 28;10:297. doi: 10.3389/fpls.2019.00297 (PMC6447655; doi:10.3389/fpls.2019.00297)

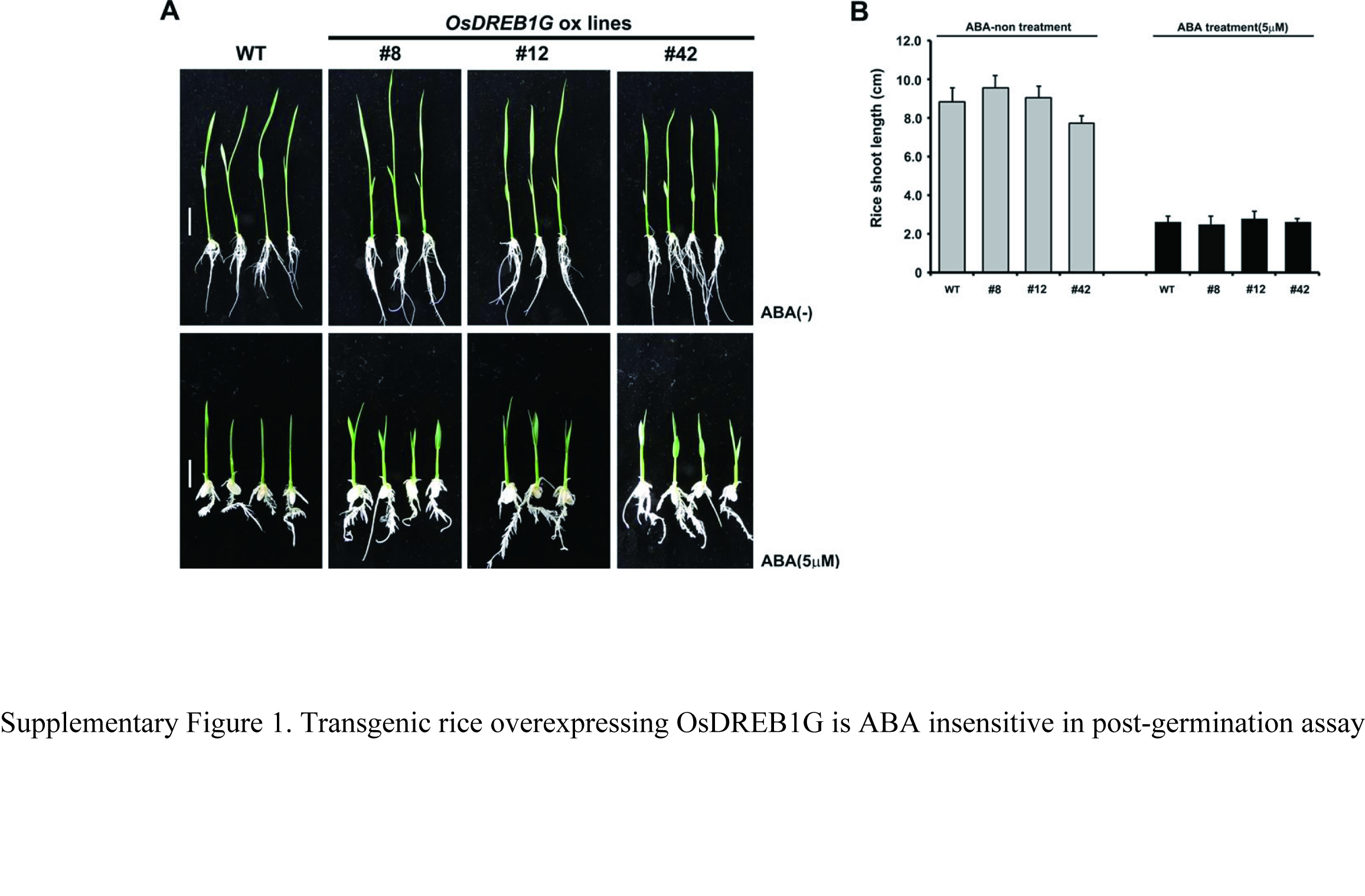

Supplement: Supplementary file 3 [file Image_1.JPEG]

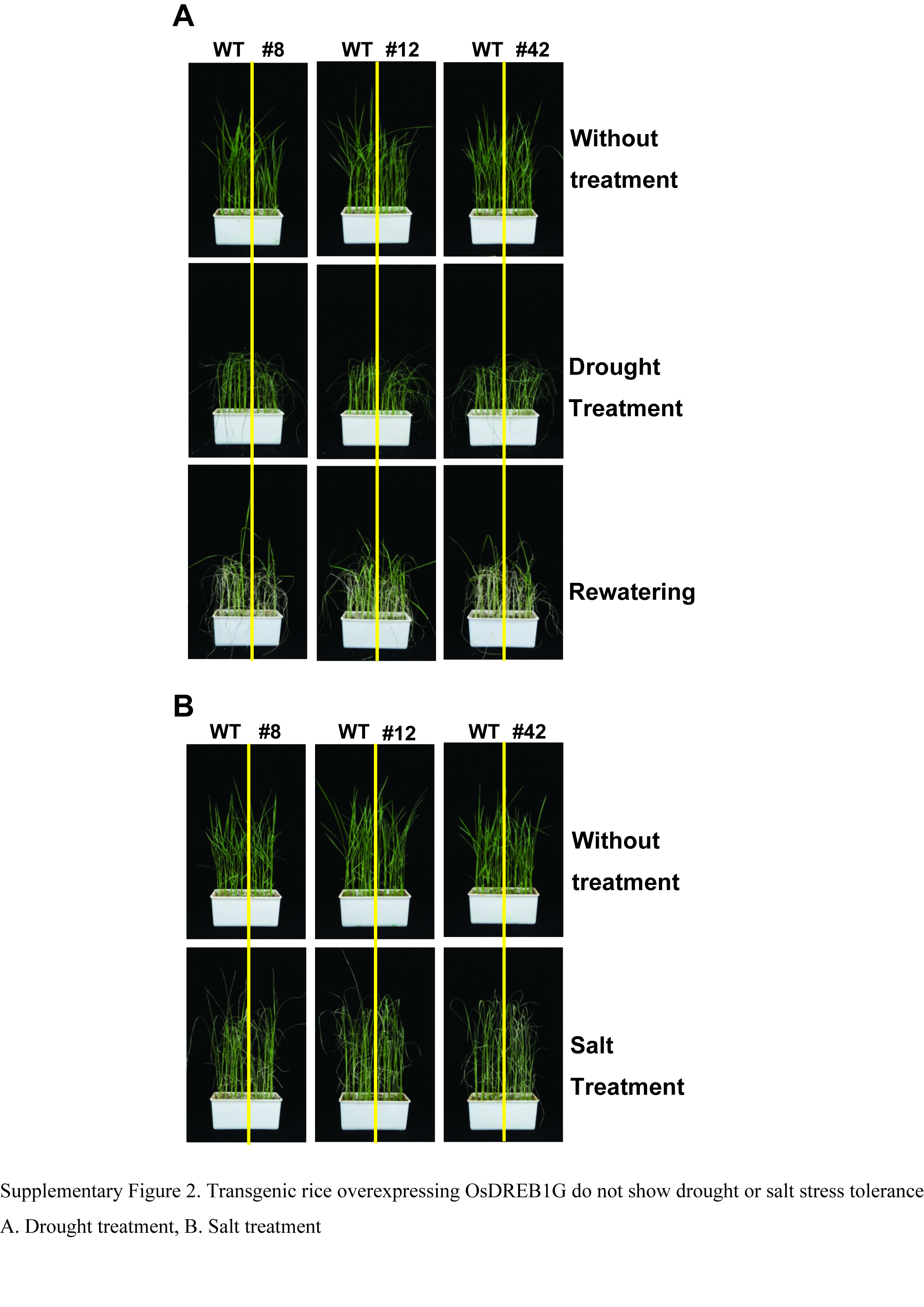

Supplement: Supplementary file 4 [file Image_2.JPEG]
